# Cardiovascular Characteristics of Zucker Fatty Diabetes Mellitus Rats, an Animal Model for Obesity and Type 2 Diabetes

**DOI:** 10.3390/ijms23084228

**Published:** 2022-04-11

**Authors:** Kosuke Otani, Hiroshi Funada, Risa Teranishi, Muneyoshi Okada, Hideyuki Yamawaki

**Affiliations:** Laboratory of Veterinary Pharmacology, School of Veterinary Medicine, Kitasato University, Towada 034-8628, Aomori, Japan; otani@vmas.kitasato-u.ac.jp (K.O.); vm17109@st.kitasato-u.ac.jp (H.F.); vm16086@st.kitasato-u.ac.jp (R.T.); mokada@vmas.kitasato-u.ac.jp (M.O.)

**Keywords:** cardiovascular dynamics, sympathetic nerve activity, obesity, diabetes, adipose tissue, kidney

## Abstract

Zucker fatty diabetes mellitus (ZFDM) rats harboring the missense mutation (fa) in a leptin receptor gene have been recently established as a novel animal model of obesity and type 2 diabetes (T2D). Here, we explored changes in cardiovascular dynamics including blood pressure and heart rate (HR) associated with the progression of obesity and T2D, as well as pathological changes in adipose tissue and kidney. There was no significant difference in systolic blood pressure (SBP) in ZFDM-Lepr^fa/fa^ (Homo) compared with ZFDM-Lepr^fa/+^ (Hetero) rats, while HR and plasma adrenaline in Homo were significantly lower than Hetero. The mRNA expression of monocyte chemotactic protein-1 in perirenal white adipose tissue (WAT) from Homo was significantly higher than Hetero. Interscapular brown adipose tissue (BAT) in Homo was degenerated and whitened. The plasma blood urea nitrogen in Homo was significantly higher than Hetero. In summary, we demonstrated for the first time that HR and plasma adrenaline concentration but not SBP in Homo decrease with obesity and T2D. In addition, inflammation occurs in WAT from Homo, while whitening occurs in BAT. Further, renal function is impaired in Homo. In the future, ZFDM rats will be useful for investigating metabolic changes associated with the progression of obesity and T2D.

## 1. Introduction

Obesity is defined as an abnormal or excessive fat accumulation that is associated with chronic metabolic diseases, including cardiovascular diseases such as heart disease and stroke [1]. In 2017, an epidemiological investigation using the Global Burden of Disease Study data reported that more than 4 million people die each year from overweight and obesity, with the highest number of deaths being cardiovascular diseases due to high body mass index (BMI) followed by diabetes and kidney disease [2]. Type 2 diabetes (T2D) is a metabolic disease in which blood glucose levels become chronically high due to decreased insulin secretion and/or insulin resistance [3]. The prevalence and incidence of T2D, which accounts for more than 90% of all diabetes cases, have been increasing worldwide in recent years [4,5]. It is known that obesity-induced insulin resistance may cause the development of T2D [6] and that 90% of children with T2D are obese at diagnosis [7]. Moreover, the complications associated with T2D, such as nephropathy [8,9] and cardiovascular diseases [10], are similarly observed with obesity. Therefore, they could be closely and multiply related each other.

Animal models of obesity and T2D have contributed greatly to basic research. There are several animal models, such as Zucker diabetic fatty (ZDF) rats [11], Otsuka Long-Evans Tokushima fatty (OLETF) rats [12], Wistar fatty rats [13], and db/db mice [14]. Similar to humans, many of these animal models exhibit cardiovascular diseases associated with obesity and diabetes [15,16]. 

Zucker fatty diabetes mellitus (ZFDM) rat strains have been established in 2008 from a heterozygous colony of Zucker fatty rats harboring the missense mutation (fa) in a leptin receptor gene [17]. ZFDM-Lepr^fa/fa^ (Homo) rats develop obesity due to overeating caused by a dysfunction of the leptin receptor. In addition, Homo rats develop T2D from a relatively early age (10 weeks old), reaching 100% incidence rate at around 20 weeks old. On the other hand, ZFDM-Lepr^fa/+^ (Hetero) rats do not develop obesity and T2D [17]. Unlike other obese and T2D model rats, Homo rats do not require high-fat diet feeding and oral glucose tolerance test to determine diabetes. Moreover, male Homo rats but not ZDF-Lepr^(fa/fa)^ rats have the reproductive ability. Then, through mating female Hetero with male Homo, there is merit in obtaining a large number of Homo pups.

In the pancreatic islets, there is a decreased insulin response to incretins, and intrinsic vulnerability of the islets may be involved in the development of T2D in Homo rats [18]. However, changes in cardiovascular dynamics including blood pressure and heart rate (HR) associated with the progression of obesity and T2D, and pathological changes in adipose tissue and kidney have not been extensively examined in ZFDM rats. In the present study, we aimed to explore them.

## 2. Results

### 2.1. The Metabolic Characteristics of ZFDM Rats

**Figure 1:**
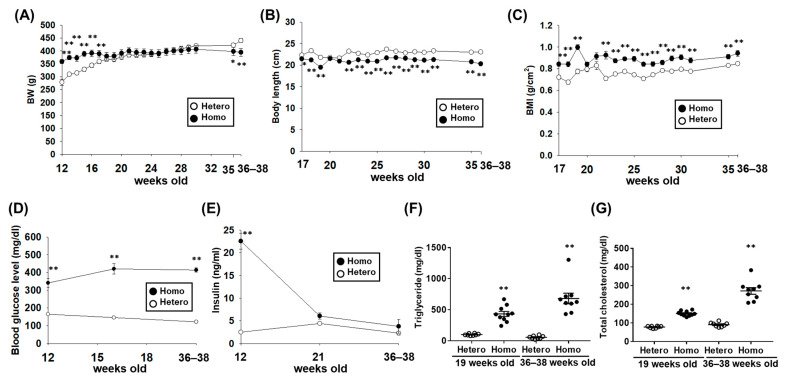
The metabolic characteristics of male Zucker fatty diabetes mellitus (ZFDM)-Lepr^fa/+^ (Hetero) and ZFDM-Lepr^fa/fa^ (Homo) rats. (**A**) Changes in body weight (BW) of ZFDM rats at 12 (*n* = 10), 13–16 (Hetero: *n* = 9, Homo: *n* = 10), 17–35 (Hetero: *n* = 8, Homo: *n* = 10), and 36–38 (Hetero: *n* = 8, Homo: *n* = 9) weeks old. (**B**,**C**) Changes in body length (**B**) and body mass index (BMI) (**C**) of ZFDM rats at 17–35 (Hetero: *n* = 8, Homo: *n* = 10) and 36–38 (Hetero: *n* = 8, Homo: *n* = 9) weeks old. BMI was calculated via dividing BW by body length squared. (**D**) Blood glucose level in ZFDM rats at 12, 16 (Hetero: *n* = 9, Homo: *n* = 10), and 36–38 (Hetero: *n* = 8, Homo: *n* = 9) weeks old was determined by an enzymatic electrode method. (**E**) The insulin concentration in heparin (1 U/mL)-anticoagulated plasma of ZFDM rats at 12, 21, and 36–38 weeks old (*n* = 5) was measured by a commercially available enzyme-linked immunosorbent assay (ELISA) kit. (**F**,**G**) The plasma triglyceride and total cholesterol levels of ZFDM rats at 19 and 36–38 weeks old (Hetero: *n* = 8, Homo: *n* = 10) were determined by a colorimetric method. Results were expressed as means ± standard error of the mean (SEM). * *p* < 0.05, ** *p* < 0.01 vs. Hetero.

Body weight (BW) in Homo at 12–17 weeks old was significantly higher than Hetero (Figure 1A, *p* < 0.01), while that in Homo was significantly lower at 35–38 weeks old (Figure 1A, Table 1, *p* < 0.05 at 35 weeks old, *p* < 0.01 at 36–38 weeks old). Body length in Homo was significantly lower than Hetero at 17–19 and 22–38 weeks old (Figure 1B, Table 1, *p* < 0.05 at 17 weeks old, *p* < 0.01 at 18, 19, and 22–38 weeks old). BMI, commonly used as an indicator of obesity [19], in Homo was significantly higher than Hetero at 17–19 and 22–38 weeks old (Figure 1C, Table 1, *p* < 0.01). The blood glucose level in Homo was significantly higher than Hetero at 12, 16, and 36–38 weeks old (Figure 1D, Table 1, *p* < 0.01). The plasma insulin concentration in Homo was significantly higher than Hetero at 12 weeks old (Figure 1E, *p* < 0.01), while no difference was observed at 21 and 36–38 weeks old (Figure 1E). The plasma levels of triglyceride and total cholesterol in Homo at 19 and 36–38 weeks old were significantly higher than Hetero (Figure 1F,G, Table 1, *p* < 0.01).

### 2.2. The Cardiovascular Dynamics of ZFDM Rats

**Figure 2:**
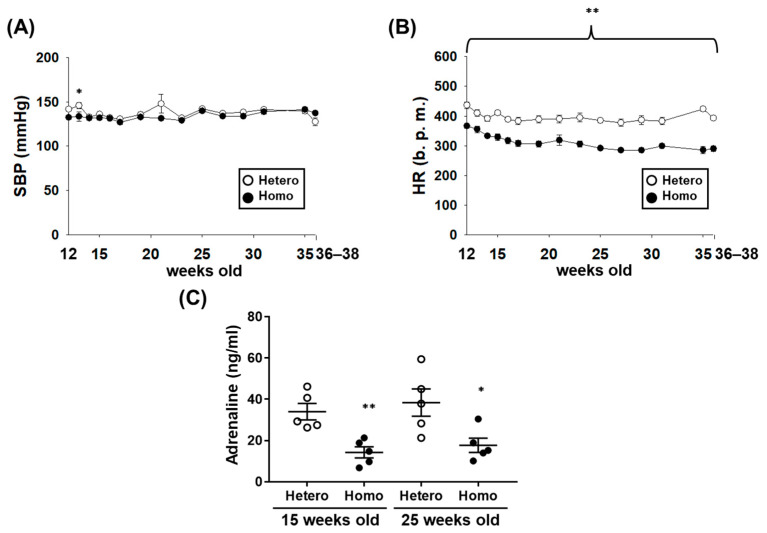
The cardiovascular dynamics of ZFDM rats. (**A**,**B**) The systolic blood pressure (SBP) (**A**) and heart rate (HR) (**B**) of ZFDM rats at 12 (*n* = 10), 13–16 (Hetero: *n* = 9, Homo: *n* = 10), 17–35 (Hetero: *n* = 8, Homo: *n* = 10), and 36–38 (Hetero: *n* = 8, Homo: *n* = 9) weeks old were measured by a tail-cuff method under conscious condition. (**C**) The adrenaline concentration in heparin (1 U/mL)-anticoagulated plasma of ZFDM rats at 15 and 25 weeks old (*n* = 5) was measured by a commercially available ELISA kit. Results were expressed as means ± SEM. * *p* < 0.05, ** *p* < 0.01 vs. Hetero.

There was almost no difference in systolic blood pressure (SBP) between Hetero and Homo (Figure 2A, Table 1). Interestingly, HR in Homo was significantly lower than Hetero (Figure 2B, Table 1, *p* < 0.01). In order to explore mechanisms of decrease in HR, we examined the plasma adrenaline concentration. It was significantly lower in Homo than Hetero at 15 and 25 weeks old (Figure 2C, *p* < 0.01 at 15 weeks old, *p* < 0.05 at 25 weeks old). The heart weight in Homo at 36–38 weeks old was slightly higher than Hetero (Table 1, *p* < 0.05).

### 2.3. Histological Analysis of Ventricles and Mesenteric Arteries from ZFDM Rats

**Figure 3:**
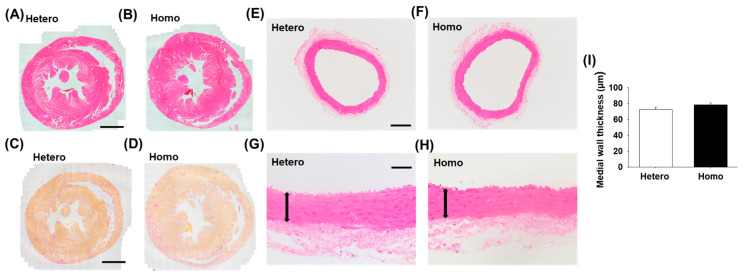
Histological analysis of ventricles and mesenteric arteries from ZFDM rats. Thin sections (4 μm) were made from paraffin-embedded isolated ventricles and mesenteric arteries from ZFDM rats at 36–38 weeks old (Hetero: *n* = 8, Homo: *n* = 9). (**A**,**B**) Representative hematoxylin and eosin (HE)-stained sections for ventricles. (**C**,**D**) Representative picrosirius red stained sections (4 µm) for ventricles. Collagen was stained in red, and cytoplasm was stained in yellow. (**E**–**H**) Representative HE-stained sections for mesenteric arteries. (**I**) Medial wall thickness of the mesenteric arteries was calculated and shown as means ± SEM. Scale bar: 2 μm (**A**,**C**), 100 μm (**E**), and 50 μm (**G**). Arrow: medial wall of the artery.

Histological changes in ventricles and mesenteric arteries in Homo at 36–38 weeks old were not observed compared with Hetero (Figure 3A,B,E,F). The accumulation of collagen was not observed in the perivascular and myocardium regions of the ventricles from both strains (Figure 3C,D). There was no significant difference in the medial wall thickness of the mesenteric arteries among the strains (Figure 3G–I). 

### 2.4. Histology and mRNA Expression in Adipocytes from ZFDM Rats

**Figure 4:**
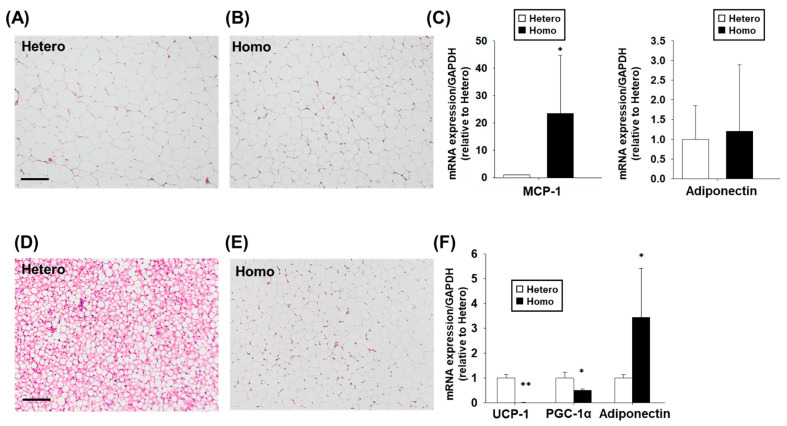
Morphology and mRNA expression in white adipose tissue (WAT) and brown adipose tissue (BAT). Thin sections (10 μm) were made from paraffin-embedded isolated perirenal adipocytes (WAT) and interscapular adipocytes (BAT) from ZFDM rats at 36–38 weeks old (Hetero: *n* = 8, Homo: *n* = 9). (**A**,**B**) Representative HE-stained sections for WAT. (**C**) The mRNA expression levels of monocyte chemotactic protein (MCP)-1 and adiponectin in WAT were measured by a reverse transcription quantitative polymerase chain reaction (RT-qPCR). The data normalized to glyceraldehyde 3-phosphate dehydrogenase (GAPDH) mRNA was shown as fold increase relative to Hetero and expressed as means ± SEM. (**D**,**E**) Representative HE-stained sections for BAT. (**F**) The mRNA expression levels of uncoupling protein (UCP)-1, peroxisome-proliferator-activated receptor γ coactivator-1α (PGC-1α), and adiponectin in BAT were measured by RT-qPCR. The data normalized to GAPDH mRNA was shown as fold increase relative to Hetero and expressed as means ± SEM. Scale bar: 200 μm (**A**,**D**). * *p* < 0.05, ** *p* < 0.01 vs. Hetero.

The adipocyte hypertrophy was not observed in perirenal white adipose tissue (WAT) from Hetero and Homo (Figure 4A,B). On the other hand, the weight of WAT in Homo at 36–38 weeks old was significantly higher than Hetero (Table 1, *p* < 0.01). The mRNA expression level of monocyte chemotactic protein (MCP)-1, a mediator of inflammation [20], in WAT from Homo was significantly higher than Hetero (Figure 4C, *p* < 0.05). There was no significant difference in the mRNA expression level of adiponectin, which is exclusively released from WAT [21] (Figure 4C). Interscapular brown adipose tissue (BAT) in Homo contained a large droplet like WAT but not a small lipid droplet (normally contained in BAT) compared with Hetero (Figure 4D,E). Uncoupling protein (UCP)-1 and peroxisome-proliferator-activated receptor γ coactivator-1α (PGC-1α) are highly expressed in BAT [22]. The mRNA expression levels of UCP-1 and PGC-1α in interscapular adipocytes from Homo were significantly lower than Hetero (Figure 4F, *p* < 0.01 in UCP-1; *p* < 0.05 in PGC-1α). We confirmed that the protein level of UCP-1 in interscapular adipocytes was lower in Homo than Hetero (data not shown, *n* = 5). On the other hand, the mRNA expression level of adiponectin (a WAT marker) in interscapular adipocytes from Homo was significantly higher than Hetero (Figure 4F, *p* < 0.05). The weight of interscapular adipocytes in Homo at 36–38 weeks old was significantly higher than Hetero (Table 1, *p* < 0.05). 

### 2.5. Changes in Weight and Biochemical Characteristics of Kidney from ZFDM Rats

The kidney weight in Homo at 36–38 weeks old was significantly higher than Hetero (Table 1, *p* < 0.01). The plasma level of blood urea nitrogen (BUN) in Homo at 36–38 weeks old was significantly higher than Hetero (Table 1, *p* < 0.05), while that of creatinine in Homo at 36–38 weeks old was significantly lower than Hetero (Table 1, *p* < 0.01).

## 3. Discussion

In this study, we investigated the cardiovascular dynamics and the changes in adipose tissues (WAT and BAT) and kidneys associated with the progression of obesity and T2D in ZFDM rats. The major findings of the present study were as follows: (1) BMI and blood glucose level in Homo were higher than Hetero at 17–38 weeks old (Figure 1C,D). Compared with Hetero, the plasma insulin concentration in Homo was significantly higher at 12 weeks old, while it was not different at 21 and 36–38 weeks old (Figure 1E). (2) There was no difference in SBP between Hetero and Homo, while HR and plasma adrenaline concentration in Homo were significantly lower than Hetero (Figure 2). No pathological changes were observed in the morphology of the ventricles and mesenteric arteries (Figure 3). (3) The weight of WAT (Table 1) and the mRNA expression level of MCP-1 (Figure 4C) in Homo were significantly higher than Hetero. The weight of adipocytes isolated from the interscapular area (normally BAT) in Homo was higher than Hetero (Table 1), and they contained a large droplet like WAT (Figure 4D,E). In addition, the mRNA expression level of UCP-1 and PGC-1α in Homo was lower, while that of adiponectin was significantly higher than Hetero (Figure 4F). (4) The plasma level of BUN in Homo was significantly higher, while that of creatinine was significantly lower than Hetero (Table 1). Collectively, the present study demonstrated for the first time that HR but not SBP in Homo decreases with obesity and T2D, perhaps due to a decrease in sympathetic nerve activity. In addition, inflammation of WAT, whitening of BAT, and impairment of renal function were observed in Homo.

In the present study, we confirmed that BW (Figure 1A), body length (Figure 1B), BMI (Figure 1C), the blood glucose level (Figure 1D), and the plasma levels of triglyceride (Figure 1F) and cholesterol (Figure 1G) in ZFDM rats (up to 21 weeks old) were almost same as those reported in the previous study [17]. On the other hand, the difference in BW between Hetero and Homo decreased after 21 weeks old, which was reversed after they were 35 weeks old. However, the BMI in Homo remained consistently higher than Hetero until they were 36–38 weeks old. The blood glucose level in Homo at 36–38 weeks old was also higher than Hetero. Therefore, it was newly confirmed that Homo was obese and hyperglycemic after 21 weeks of age. The plasma insulin concentration in Homo was significantly higher than Hetero at 12 weeks old, while it was not different at 21 and 36–38 weeks old (Figure 1E). Therefore, it is assumed that insulin secretion decreased in Homo after 12 weeks of age. It was reported that pancreatic islet atrophy was observed in Homo after the age of 12 weeks old [18], which we confirmed in Homo at 36–38 weeks old (Figure S2, see Supplementary Materials). 

In the present study, the plasma adrenaline concentration (referred to as sympathetic nerve activity) [23] in Homo was significantly lower than Hetero (Figure 2C). The blood pressure of leptin-overexpressed mice was elevated by the increase in sympathetic nerve activity [24]. Further, microinjection of leptin into the arcuate nucleus of brain activated sympathetic nerve activity in SD rats [25]. ZFDM rats have a missense mutation in *Lepr* gene [17] that caused the impaired signal transduction mediated by leptin receptor [26]. Then, it is suggested that the impairment of leptin receptor signal in the brain would mediate the decrease in sympathetic nerve activity in Homo. The decrease in the plasma adrenaline concentration in Homo (Figure 2C) may mediate the decrease in SBP as well as HR. However, there was no difference in SBP between Hetero and Homo (Figure 2A), nor was there any thickening of the vessels associated with the changes in blood pressure (Figure 3E–I). Further research focusing on vascular reactivity that affects peripheral blood pressure is needed to explore the reason for this discrepancy.

Diabetes increases the risk of cardiomyopathy [27]. As shown in Figure 3A–D, however, hypertrophy and fibrosis were not observed in the hearts from Homo rats. Diabetic cardiomyopathy is a pathological condition of heart failure characterized by hypertrophy and fibrosis of the heart [27]. Therefore, it seems unlikely that ZFDM rats would exhibit diabetic cardiomyopathy. In the electrocardiogram analysis, the pathological changes except for the decrease in heart rate of Homo (Table S1) were not observed, supporting this idea.

Cardiac dysfunction is often observed in human T2D patients at later stages [27]. In this regard, comparison to other existing T2D models and limitation of ZFDM rats as a model for T2D cardiac complication should be noted. Although ejection fraction (EF) in ZDF rats at 14 weeks old seemed to be lower than control lean rats, it is suggested that certain correlation did not exist between diabetes and impaired cardiac function [16]. Another report demonstrated in ZDF rats that fractional shortening and EF were rather increased with the existence of cardiomyocyte hypertrophy at 19 weeks old, indicating that the cardiac dysfunction was not obvious [28]. Further, dysfunction of the left ventricles including impaired EF was not observed in ZDF rats at 37 weeks old [29]. Perivascular and interstitial fibrosis in the ventricles was observed in OLETF rats, another obese and T2D model, while there was no significant difference in heart function between OLETF rats and control Long-Evans Tokushima Otsuka rats at 40 [30] and 62 [15] weeks old. In addition, although HR of diabetic Goto-Kakizaki (GK) rats was decreased compared with non-diabetic control Wistar rats [31], the heart function was normal [32]. Although we did not perform the detailed functional analysis using echocardiography, hypertrophy and fibrosis were not observed in the hearts from Homo rats (Figure 3A–D), suggesting that ZFDM rat model might not exactly reflect the cardiac complications observed in human T2D patients at later stages. On the other hand, it is demonstrated that heart failure induced by coronary artery ligation was exacerbated in GK rats compared with Wistar Kyoto rats [32]. Then, the diabetic cardiomyopathy with cardiac dysfunction could be induced by the additional treatment in ZFDM rats.

In human obesity, chronic inflammation, fibrosis [33], hypertrophy, and proliferation of adipocytes [34] are observed in WAT. In the present study, there was no adipocyte hypertrophy in the perirenal WAT in Homo compared with Hetero (Figure 4A,B), while the mRNA expression level of MCP-1, a mediator of WAT inflammation, in Homo was significantly higher than Hetero (Figure 4C). It is thus suggested that inflammation occurred in WAT from Homo with obesity, which may mediate the insulin resistance. Adipocytes isolated from interscapular area (normally BAT) in Homo contained a large droplet like WAT but not a small lipid droplet (normally contained in BAT) compared with Hetero (Figure 4D,E). Similar to the present results, BAT in db/db mice, which are also leptin receptor-deficient strains, contained a large droplet like WAT [35]. The mRNA expression level of UCP-1 and PGC-1*α* was significantly lower, while that of adiponectin (normally released by WAT) was significantly higher in adipocytes isolated from interscapular area in Homo compared with Hetero (Figure 4F). Since UCP-1 [22] and PGC-1α [36] are the BAT-specific marker proteins, it is suggested that the adipocytes isolated from the interscapular area in Homo degenerated and were replaced by WAT. It is widely known that BAT is a thermogenic organ [37]. It is also known that BAT is involved in the homeostasis of glucose metabolism, since it was reported that BAT-positive humans consume more whole-body glucose after insulin administration than BAT-negative humans [38]. Thus, in Homo, the degeneration of BAT and its replacement by WAT may cause insulin resistance, exacerbating the pathogenesis of diabetes.

The kidney disease is another major complication of diabetes [39]. Glomerulopathy, tubulointerstitial fibrosis, and infiltration of inflammatory cells were observed in human T2D patients [39]. In the present study, the weight of kidney in Homo at 36–38 weeks old was significantly higher than Hetero (Table 1). The plasma level of BUN in Homo was significantly higher than Hetero (Table 1). It was reported that ZDF rats at 8 months old showed similarly elevated BUN level and severely impaired renal function [40]. In ZDF rats, the damage to glomerulus was occurring from 20 weeks old [41] and the tubulointerstitial fibrosis occurred at 21 weeks old [42]. In OLETF rats, the mesangial expansion was occurring from 22 weeks old and the glomerular sclerosis occurred at 46 weeks old [43]. Further, it is demonstrated that the tubular basement membrane was impaired in OLETF rats at 62 weeks old [44]. Several features of kidney injuries including the necrosis-like morphology in proximal tubule and the tubulointerstitial fibrosis were observed in Homo at 36–38 weeks old (Figure S1). These results suggest that the impairment of renal function might occur in Homo rats, while further investigations including renal function and characterizations at molecular levels are required. We also showed that the plasma level of creatinine in Homo was significantly lower than Hetero (Table 1). Human diabetes patients also showed lower plasma level of creatinine due to a lower muscle mass [45]. Therefore, the muscle mass loss associated with the diabetes may occur in Homo as it did in humans. In the present study, after 25 weeks of age, when the pathogenesis of diabetes was more advanced, the BW in Homo was lower than Hetero, suggesting that muscle mass loss was occurring in Homo.

In conclusion, we demonstrated for the first time that HR but not SBP in Homo decreases with obesity and T2D, perhaps due to a decrease in sympathetic nerve activity. In addition, we demonstrated that inflammation occurs in the WAT from Homo, while the whitening occurs in BAT. Further, renal function is impaired in Homo. In the future, ZFDM rats will be useful not only as a model for obesity and T2D, but also for investigating changes in cardiovascular dynamics, adipocytes, and kidney function associated with the progression of obesity and T2D.

## 4. Materials and Methods

### 4.1. Animals

All animal studies were approved by the President of the Kitasato University through the judgment by Institutional Animal Care and Use Committee of the Kitasato University (Approval No. 20-004). Male ZFDM rats (Hoshino Laboratory Animals, Ibaraki, Japan) at 12–38 weeks old were cared in accordance with the institutional guideline for animal care and treatment. After all the measurements were performed, the rats were euthanized by exsanguination under deep urethane (1.5 g/kg, i.p.) anesthesia. Then, the heart, kidney, WAT, BAT, and superior mesenteric artery were isolated from 36–38 weeks old ZFDM rats (Hetero: *n* = 8, Homo: *n* = 9). They were immediately frozen with a liquid nitrogen and preserved at −80 °C for reverse transcription quantitative polymerase chain reaction (RT-qPCR) analysis, or fixed with 4% paraformaldehyde for a histological analysis.

### 4.2. Calculation of BMI

We measured BW of ZFDM rats at 12 (*n* = 10), 13–16 (Hetero: *n* = 9, Homo: *n* = 10), 17–35 (Hetero: *n* = 8, Homo: *n* = 10), and 36–38 (Hetero: *n* = 8, Homo: *n* = 9) weeks old. The body length of ZFDM rats was measured at 17–35 (Hetero: *n* = 8, Homo: *n* = 10) and 36–38 (Hetero: *n* = 8, Homo: *n* = 9) weeks old once a week. The BMI was calculated via dividing BW by body length squared [46]. 

### 4.3. Blood Glucose Measurement

Blood glucose level was determined using One touch ultra (Johnson and Johnson, Tokyo, Japan), following the manufacturer’s instructions. The blood was collected from tail vein of ZFDM rats at 12 (*n* = 10), 16 (Hetero: *n* = 9, Homo: *n* = 10), and 36–38 (Hetero: *n* = 8, Homo: *n* = 9) weeks old under a conscious condition.

### 4.4. Plasma Insulin, Triglyceride, Total Cholesterol, BUN, and Creatinine Measurements 

The blood was drawn via a tail vein of ZFDM rats at 12, 19, and 21 weeks old under a conscious condition, or via a posterior vena cava of urethane (1.5 g/kg, i. p.)-anesthetized ZFDM rats at 36–38 weeks old. The collected blood was mixed with heparin (final concentration of 1 U/mL) and centrifuged (1000× *g*, 10 min, room temperature). The supernatant was collected as plasma sample. The insulin concentration in plasma of ZFDM rats at 12, 21, and 36–38 weeks old (*n* = 5) was measured by a LBIS Rat-T Insulin enzyme-linked immunosorbent assay (ELISA) kit (Fuji Film Wako, Osaka, Japan), following the manufacturer’s instructions. The triglyceride, total cholesterol, BUN, and creatinine concentrations in plasma of ZFDM rats at 19 (Hetero: *n* = 8, Homo: *n* = 10) and/or 36–38 (Hetero: *n* = 8, Homo: *n* = 9) weeks old were measured using a Dimension RxL Max Integrated Chemistry System (Siemens Healthineers, Erlangen, Germany), following the manufacturer’s instructions [47].

### 4.5. Measurement of SBP and HR

We measured SBP and HR of ZFDM rats at 12 (*n* = 10), 13–16 (Hetero: *n* = 9, Homo: *n* = 10), 17–35 (Hetero: *n* = 8, Homo: *n* = 10), and 36–38 (Hetero: *n* = 8, Homo: *n* = 9) weeks old by a tail-cuff system (BP-98AL; Softron, Tokyo, Japan) under conscious condition as described previously [48].

### 4.6. Plasma Adrenaline Measurement

The adrenaline concentration in plasma of ZFDM rats at 15 and 25 weeks old (*n* = 5) was measured using an Epinephrine/Norepinephrine ELISA kit (KA1877; Abnova, Taipei, Taiwan), following the manufacturer’s instructions. 

### 4.7. Histological Analysis

Ventricles, mesenteric arteries, WAT, and BAT from ZFDM rats at 36–38 weeks old fixed with 4% paraformaldehyde in phosphate buffer were embedded in pathoprep 568 (Fuji Film Wako, Osaka, Japan) and sectioned (ventricles and mesenteric artery in 4 μm, WAT and BAT in 10 μm). Hematoxylin and eosin (HE) or Picrosirius red staining was performed as described previously [48]. The images were obtained using a light microscope (BX-51; OLYMPUS, Tokyo, Japan) equipped with a microscope digital camera (DP74; OLYMPUS, Tokyo, Japan).

### 4.8. RT-qPCR Analysis

qPCR was performed using THUNDERBIRD SYBR qPCR MIX (TOYOBO, Osaka, Japan) as described previously [49]. WAT and BAT from ZFDM rats at 36–38 weeks old were homogenized by using a prechilled mortar and pestle in liquid nitrogen. Total RNA was extracted from the homogenized tissues using TRI Reagent (Cosmo Bio Co., Tokyo, Japan). The cDNA was obtained from the total RNA using ReverTra Ace qPCR RT Master Mix with a gDNA Remover (TOYOBO, Osaka, Japan). After initial activation at 95 °C (1 min), the amplification was run for 50 cycles in MCP-1, UCP-1, PGC-1*α*, adiponectin, and glyceraldehyde-3-phosphate dehydrogenase (GAPDH) at 95 °C (15 s), 60 °C (30 s), and 60 °C (30 s). The primer sequences were shown in Table 2. The mRNA expression level to GAPDH was calculated from cycle threshold (Cq) value by a ΔΔCq method and was shown as fold increase relative to Hetero. 

### 4.9. Statistical Analysis

Data were shown as mean ± means ± standard error of the mean (SEM). Statistical evaluations were performed by Student’s *t*-test. A value of *p* < 0.05 was considered statistically significant.

## Figures and Tables

**Table 1:** Physical and biochemical parameters in Zucker fatty diabetes mellitus (ZFDM)-Lepr^fa/+^ (Hetero) and ZFDM-Lepr^fa/fa^ (Homo) rats at 36–38 weeks old. BW: body weight, BMI: body mass index, SBP: systolic blood pressure, HR: heart rate, BUN: blood urea nitrogen. Results were expressed as means ± standard error of the mean (Hetero: *n* = 8, Homo: *n* = 9). * *p* < 0.05, ** *p* < 0.01 vs. Hetero.

	Hetero (*n* = 8)	Homo (*n* = 9)
BW (g)	450.2 ± 8.8	389.6 ± 14.7 **
Body length (cm)	23.1 ± 0.1	20.3 ± 0.2 **
BMI (g/cm^2^)	0.85 ± 0.01	0.94 ± 0.03 **
Blood glucose level (mg/dL)	162.5 ± 8.2	504.3 ± 20.0 **
Triglyceride (mg/dL)	56.5 ± 8.8	681.2 ± 85.2 **
Total cholesterol (mg/dL)	90.9 ± 4.3	271.8 ± 18.7 **
Heart/BW (mg/g)	3.2 ± 0.1	3.5 ± 0.1 *
SBP (mmHg)	130.5 ± 1.9	127.4 ± 4.4
HR (b. p. m.)	393.8 ± 8.6	282.9 ± 11.2 **
Perirenal adipocytes/BW (mg/g)	13.8 ± 1.0	48.5 ± 3.5 **
Interscapular adipocytes/BW (mg/g)	1.5 ± 0.1	2.1 ± 0.3 *
Kidney/BW (mg/g)	5.8 ± 0.2	8.6 ± 0.2 **
BUN (mg/dL)	24.1 ± 0.7	27.4 ± 0.8 *
Creatinine (mg/dL)	0.65 ± 0.03	0.33 ± 0.01 **

**Table 2:** Primer sequences for quantitative polymerase chain reaction. MCP-1: monocyte chemotactic protein-1, UCP-1: uncoupling proein-1, PGC-1*α*: peroxisome proliferator activated receptor γ coactivator-1α, GAPDH: glyceraldehyde 3-phosphate dehydrogenase.

Products		Primer Sequences	Accession Number
MCP-1	Reverse	5′-CCAATGAGTCGGCTGGAGAACT-3′	NM_031530.1
	Forward	5′-AGTGCTTGAGGTGGTTGTGGAA-3′	
UCP-1	Reverse	5′-GCCTCTACGATACGGTCCAA-3′	NM_012682.2
	Forward	5′-CTGACCTTCACCACCTCTGT-3′	
PGC-1*α*	Reverse	5′-ACCCACAGGATCAGAACAAACC-3′	NM_031347.1
	Forward	5′-GACAAATGCTCTTTGCTTTATTGC-3′	
Adiponectin	Reverse	5′-GAAGGGAGACGCAGGTGTTC-3′	NM_144744.3
	Forward	5′-GGGAACATTGGGGACAGTGA-3′	
GAPDH	Reverse	5′-GAAGACGCCAGTAGACTCCA-3′	NM_017008.4
	Forward	5′-GAGAATGGGAAGCTGGTCAT-3′	

## Data Availability

Not applicable.

## Supplementary Materials

The following supporting information can be downloaded at: https://www.mdpi.com/article/10.3390/ijms23084228/s1.

## References

[1] WHO Obesity. https://www.who.int/health-topics/obesity#tab=tab_1.

[2] Dai H., Alsalhe T.A., Chalghaf N., Riccò M., Bragazzi N.L., Wu J. (2020). The Global Burden of Disease Attributable to High Body Mass Index in 195 Countries and Territories, 1990–2017: An Analysis of the Global Burden of Disease Study. PLoS Med..

[3] Galicia-Garcia U., Benito-Vicente A., Jebari S., Larrea-Sebal A., Siddiqi H., Uribe K.B., Ostolaza H., Martín C. (2020). Pathophysiology of Type 2 Diabetes Mellitus. Int. J. Mol. Sci..

[4] Chatterjee S., Khunti K., Davies M.J. (2017). Type 2 diabetes. Lancet.

[5] Wild S., Roglic G., Green A., Sicree R., King H. (2004). Global Prevalence of Diabetes: Estimates for the year 2000 and projections for 2030. Diabetes Care.

[6] Kahn B.B., Flier J.S. (2000). Obesity and insulin resistance. J. Clin. Investig..

[7] Reinehr T. (2013). Type 2 diabetes mellitus in children and adolescents. World J. Diabetes.

[8] Reddy M.A., Park J.T., Natarajan R. (2013). Epigenetic Modifications in the Pathogenesis of Diabetic Nephropathy. Semin. Nephrol..

[9] de Boer I.H., Rue T.C., Hall Y.N., Heagerty P.J., Weiss N.S., Himmelfarb J. (2011). Temporal Trends in the Prevalence of Diabetic Kidney Disease in the United States. JAMA.

[10] Viigimaa M., Sachinidis A., Toumpourleka M., Koutsampasopoulos K., Alliksoo S., Tiinatma T. (2020). Macrovascular Complications of Type 2 Diabetes Mellitus. Curr. Vasc. Pharmacol..

[11] Peterson R.G., Shaw W.N., Neel M.A., Little L.A., Eichberg J. (1990). Zucker Diabetic Fatty Rat as a Model for Non-insulin-dependent Diabetes Mellitus. ILAR J..

[12] Kawano K., Hirashima T., Mori S., Saitoh Y., Kurosumi M., Natori T. (1992). Spontaneous Long-Term Hyperglycemic Rat with Diabetic Complications. Diabetes.

[13] Ikeda H., Shino A., Matsuo T., Iwatsuka H., Suzuoki Z. (1981). A New Genetically Obese-Hyperglycemic Rat (Wistar Fatty). Diabetes.

[14] Hummel K.P., Dickie M.M., Coleman D.L. (1966). Diabetes, a New Mutation in the Mouse. Science.

[15] Saito F., Kawaguchi M., Izumida J., Asakura T., Maehara K., Maruyama Y. (2003). Alteration in haemodynamics and pathological changes in the cardiovascular system during the development of Type 2 diabetes mellitus in OLETF rats. Diabetologia.

[16] Marsh S.A., Powell P.C., Agarwal A., Dell’Italia L.J., Chatham J.C. (2007). Cardiovascular dysfunction in Zucker obese and Zucker diabetic fatty rats: Role of hydronephrosis. Am. J. Physiol. Heart Circ. Physiol..

[17] Yokoi N., Hoshino M., Hidaka S., Yoshida E., Beppu M., Hoshikawa R., Sudo K., Kawada A., Takagi S., Seino S. (2013). A Novel Rat Model of Type 2 Diabetes: The Zucker Fatty Diabetes Mellitus ZFDM Rat. J. Diabetes Res..

[18] Gheni G., Yokoi N., Beppu M., Yamaguchi T., Hidaka S., Kawabata A., Hoshino Y., Hoshino M., Seino S. (2015). Characterization of the Prediabetic State in a Novel Rat Model of Type 2 Diabetes, the ZFDM Rat. J. Diabetes Res..

[19] Blackburn H., Jacobs D. (2014). Commentary: Origins and evolution of body mass index (BMI): Continuing saga. Int. J. Epidemiol..

[20] Wozniak S.E., Gee L.L., Wachtel M.S., Frezza E.E. (2009). Adipose Tissue: The New Endocrine Organ? A Review Article. Dig. Dis. Sci..

[21] Bełtowski J. (2003). Adiponectin and resistin-new hormones of white adipose tissue. Med. Sci. Monit..

[22] Chabowska-Kita A., Kozak L.P. (2016). The critical period for brown adipocyte development: Genetic and environmental influences. Obesity.

[23] Pagani M., Malliani A. (2000). Interpreting oscillations of muscle sympathetic nerve activity and heart rate variability. J. Hypertens..

[24] Aizawa-Abe M., Ogawa Y., Masuzaki H., Ebihara K., Satoh N., Iwai H., Matsuoka N., Hayashi T., Hosoda K., Inoue G. (2000). Pathophysiological role of leptin in obesity-related hypertension. J. Clin. Investig..

[25] Rahmouni K., Morgan D.A. (2007). Hypothalamic arcuate nucleus mediates the sympathetic and arterial pressure responses to leptin. Hypertension.

[26] Yamashita T., Murakami T., Iida M., Kuwajima M., Shima K. (1997). Leptin Receptor of Zucker Fatty Rat Performs Reduced Signal Transduction. Diabetes.

[27] Ritchie R.H., Abel B.D. (2020). Basic Mechanisms of Diabetic Heart Disease. Circ. Res..

[28] Fredersdorf S., Thumann C., Ulucan C., Griese D.P., Luchner A., Riegger G.A., Kromer E.P., Weil J. (2004). Myocardial hypertrophy and enhanced left ventricular contractility in Zucker diabetic fatty rats. Cardiovasc. Pathol..

[29] Schäfer S., Huber J., Wihler C., Rütten H., Busch A.E., Linz W. (2006). Impaired left ventricular relaxation in type 2 diabetic rats is related to myocardial accumulation of N(epsilon)-(carboxymethyl) lysine. Eur. J. Heart Fail..

[30] Makino N., Maeda T., Oyama J., Higuchi Y., Mimori K. (2009). Improving insulin sensitivity via activation of PPAR-γ increases telomerase activity in the heart of OLETF rats. Am. J. Physiol. Heart Circ. Physiol..

[31] Howarth F.C., Jacobson M., Shafiullah M., Adeghate E. (2008). Long-term effects of type 2 diabetes mellitus on heart rhythm in the Goto-Kakizaki rat. Exp. Physiol..

[32] Chandler M.P., Morgan E.E., McElfresh T.A., Kung T.A., Rennison J.H., Hoit B.D., Young M.E. (2007). Heart failure progression is accelerated following myocardial infarction in type 2 diabetic rats. Am. J. Physiol. Heart Circ. Physiol..

[33] Hepler C., Gupta R.K. (2017). The Expanding Problem of Adipose Depot Remodeling and Postnatal Adipocyte Progenitor Recruitment. Mol. Cell Endocrinol..

[34] Hirsch J., Han P.W. (1969). Cellularity of rat adipose tissue: Effects of growth, starvation, and obesity. J. Lipid Res..

[35] Kotzbeck P., Giordano A., Mondini E., Murano I., Severi I., Venema W., Cecchini M.P., Kershaw E.E., Barbatelli G., Haemmerle G. (2018). Brown adipose tissue whitening leads to brown adipocyte death and adipose tissue inflammation. J. Lipid Res..

[36] Bost F., Kaminski L. (2019). The metabolic modulator PGC-1α in cancer. Am. J. Cancer Res..

[37] Fenzl A., Kiefer F.W. (2014). Brown. adipose tissue and thermogenesis. Horm. Mol. Biol. Clin. Investig..

[38] Chondronikola M., Volpi E., Børsheim E., Porter C., Annamalai P., Enerbäck S., Lidell M.E., Saraf M.K., Labbe S.M., Hurren N.M. (2014). Brown Adipose Tissue Improves Whole-Body Glucose Homeostasis and Insulin Sensitivity in Humans. Diabetes.

[39] Thomas M.C., Brownlee M., Susztak K., Sharma K., Jandeleit-Dahm K.A.M., Zoungas S., Rossing P., Groop P.H., Cooper M.E. (2015). Diabetic kidney disease. Nat. Rev. Dis. Primes.

[40] Siwy J., Zoja C., Klein J., Benigni A., Mullen W., Mayer B., Mischak H., Jankowski J., Stevens R., Vlahou A. (2012). Evaluation of the Zucker diabetic fatty (ZDF) rat as a model for human disease based on urinary peptidomic profiles. PLoS ONE.

[41] Togashi Y., Miyamoto Y. (2013). Urinary cystatin C as a biomarker for diabetic nephropathy and its immunohistochemical localization in kidney in Zucker diabetic fatty (ZDF) rats. Exp. Toxicol. Pathol..

[42] Miyata K., Ohashi N., Suzaki Y., Katsurada A., Kobori H. (2008). Sequential activation of the reactive oxygen species/angiotensinogen/renin-angiotensin system axis in renal injury of type 2 diabetic rats. Clin. Exp. Pharmacol. Physiol..

[43] Fukuzawa Y., Watanabe Y., Inaguma D., Hotta N. (1996). Evaluation of glomerular lesion and abnormal urinary findings in OLETF rats resulting from a long-term diabetic state. J. Lab. Clin. Med..

[44] Yabuki A., Tahara T., Taniguchi K., Matsumoto M., Suzuki S. (2006). Neuronal nitric oxide synthase and cyclooxygenase-2 in diabetic nephropathy of type 2 diabetic OLETF rats. Exp. Anim..

[45] Kashima S., Inoue K., Matsumoto M. (2021). Low creatinine levels in diabetes mellitus among older individuals: The Yuport Medical Checkup Center Study. Sci. Rep..

[46] Engelbregt M.J.T., van Weissenbruch M.M., Popp-Snijders C., Lips P., Delemarre-van de Waal H.A. (2001). Body Mass Index, Body Composition, and Leptin at Onset of Puberty in Male and Female Rats after Intrauterine Growth Retardation and after Early Postnatal Food Restriction. Pediatr. Res..

[47] Sugisawa R., Hiramoto E., Matsuoka S., Iwai S., Takai R., Yamazaki T., Mori N., Okada Y., Takeda N., Yamamura K. (2016). Impact of feline AIM on the susceptibility of cats to renal disease. Sci. Rep..

[48] Otani K., Yokoya M., Kodama T., Hori K., Matsumoto K., Okada M., Yamawaki H. (2018). Plasma exosomes regulate systemic blood pressure in rats. Biochem. Biophys. Res. Commun..

[49] Sugiyama A., Okada M., Yamawaki H. (2020). Canstatin suppresses isoproterenol-induced cardiac hypertrophy through inhibition of calcineurin/nuclear factor of activated T-cells pathway in rats. Eur. J. Pharmacol..

