# Supplementary material for: Cardiovascular Characteristics of Zucker Fatty Diabetes Mellitus Rats, an Animal Model for Obesity and Type 2 Diabetes
Source: Int J Mol Sci. 2022 Apr 11;23(8):4228. doi: 10.3390/ijms23084228 (PMC9027163; doi:10.3390/ijms23084228)
Supplement: Supplementary file 1 [file ijms-23-04228-s001.zip › ijms-1659837-supplementary.pdf]

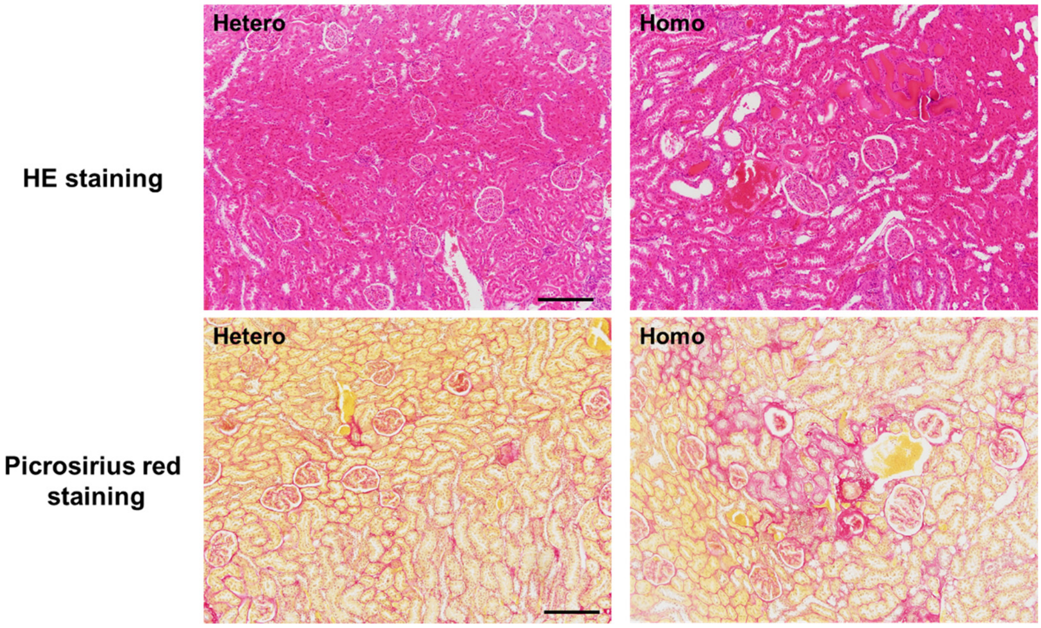

Figure S1. Histological analysis of kidney from Zucker Fatty Diabetes Mellitus (ZFDM)-Lepr$^{fa/+}$ (Hetero) and ZFDM-Lepr$^{fa/fa}$ (Homo) rats. Thin sections (4 μm) were made from paraffin-embedded isolated kidney from ZFDM rats at 36–38 weeks old (Hetero: n = 8, Homo: n = 10). Representative hematoxylin and eosin (HE) and picrosirius red stained sections were shown. In the picrosirius red staining, collagen was stained in red and cytoplasm was stained in yellow. Scale bar: 100 μm.

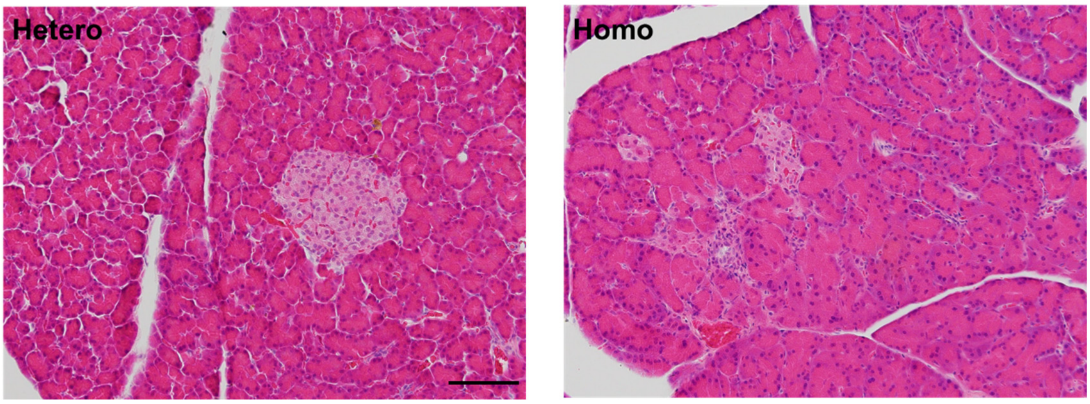

Figure S2. Histological analysis of pancreas from ZFDM rats. Thin sections (4 μm) were made from paraffin-embedded isolated pancreas from ZFDM rats at 36–38 weeks old (Hetero: *n* = 6, Homo: *n* = 7). Representative HE stained sections were shown. Scale bar: 100 μm.

Table S1. The electrocardiogram analysis of Zucker Fatty Diabetes Mellitus (ZFDM)-$Lepr^{fa/+}$ (Hetero) and ZFDM-$Lepr^{fa/fa}$ (Homo) at 20 weeks old (Hetero: $n = 8$, Homo: $n = 10$). The rats were anesthetized by an isoflurane (2.5%) inhalation. Electrocardiogram was recorded in lead II configuration. Results were expressed as means ± standard error of the mean. $^{**}p < 0.01$ vs. Hetero.

	**Hetero**	**Homo**
R interval (ms)	214.9 ± 5.6	236.2 ± 3.8**
Heart rates (b. p. m.)	280.6 ± 7.5	254.7 ± 4.0**
PR interval (ms)	53.1 ± 1.1	50.2 ± 1.9
P duration (ms)	16.0 ± 1.7	22.6 ± 2.8
QRS interval (ms)	16.0 ± 0.7	16.7 ± 1.4
P amplitude (μV)	25.8 ± 3.3	33.9 ± 13.1
R amplitude (μV)	377.1 ± 40.0	395.2 ± 69.4
